# Pain as a Correlate of Hoarding in Neurocognitive Conditions

**DOI:** 10.1002/alz70857_102330

**Published:** 2025-12-24

**Authors:** Yiorgos Antoniadis, Peter S. Pressman, David Arciniegas, Julia C Schaffer

**Affiliations:** ^1^ St. George's University, True Blue, Saint George, Grenada; ^2^ Layton Healthy Aging and Alzheimer's Disease Research Center, Portland, OR, USA; ^3^ University of Colorado Anschutz Medical Campus, Aurora, CO, USA; ^4^ University of Colorado School of Medicine, Aurora, CO, USA

## Abstract

**Background:**

Hoarding is a complex behavior associated with poor quality of life for both patients and caregivers. Although the link between hoarding behavior and comorbid disorders such as depression, anxiety and chronic pain have been suggested, these associations have been underexplored among individuals with neurological conditions. We aim to investigate the association between reported hoarding behavior and comorbidities in patients with neurocognitive conditions.

**Method:**

We administered a single‐item hoarding screen (SIHS) and Neuropsychiatric Inventory Questionnaire (NPIQ) to be filled by caregivers, as well as Patient Health Questionnaire (PHQ‐9), Generalized Anxiety Questionnaire (GAD‐7), and PROMIS‐10 questionnaire including a 1‐10 pain scale which were self‐reported by clinic patients with neurocognitive conditions during their initial healthcare visit. We then investigated correlations between the SIHS and the additional measures in each assessment tool.

**Result:**

A mixed effects analysis, accounting for repeated measures, revealed that positive hoarding screens were linked to significantly higher pain ratings with an estimated increase of approximately 3 points (β = 3.26, *p* =  0.004, CI [1.05, 5.47], *R* ^2^ = 0.13). This association remained after controlling for sex, age, and disease severity. Notably, no significant correlations were found between hoarding and measures of anxiety or negative mood symptoms on either patient or caregiver reported measures.

**Conclusion:**

Our findings indicate a relationship between hoarding behaviors and increased pain in patients with neurocognitive conditions, aligning with recent population‐based literature showing increased chronic pain in hoarding disorder. An absence of correlation with negative mood symptoms or anxiety, commonly observed in studies with other populations, suggest a distinct mechanism in these neurocognitively impaired populations. Further studies should consider investigating shared neurobiological substrates in pain processing and executive function, particularly in the anterior cingulate cortex and insula to gain a better understanding of these associations.

